# Deep Phenotyping of Musicians’ Upper Limb Dystonia

**DOI:** 10.5334/tohm.1044

**Published:** 2025-07-17

**Authors:** Steven J. Frucht

**Affiliations:** 1 Professor of Neurology, NYU Grossman School of Medicine, 222 East 41^st^ Street, 13^th^ floor, New York, NY 10017, US

**Keywords:** musician, dystonia, writer’s cramp, botulinum toxin

## Abstract

**Background:**

Focal task-specific dystonia of the musicians’ arm (FTSDma) is an unusual and challenging disorder, often causing significant disability with loss of performing careers. The etiology and optimal management of this disorder remains unclear.

**Methods:**

We reviewed records and videos of 173 patients with FTSDma, 50 patients with writer’s cramp (WC), and 16 with other forms of arm dystonia (OD), evaluated by a single examiner in clinical practice over a 25-year period. Detailed analysis of clinical features and videotaped examinations in slow motion (what we call “deep phenotyping”) allowed separation of patients into four categories: “precision-grip” dystonia (groups I and III); “power-grip” dystonia (group II); and “proximal dystonia” (group IV). We compared these results to deep phenotyping of patients with FTSDma, WC and OD patients reported in the literature.

**Results:**

FTSDma usually affects men, involves the right hand, and begins in the fourth decade. The precision hand of pianists and guitarists (digits 1, 2, 3) was preferentially affected in the right arm, and many of the remaining patients involved the power hand of either arm (digits 3, 4, 5). The dystonic phenotype of the bow arm of string players and drumming arm of stick drummers bore striking resemblance to WC and racquet dystonia, almost always involving the wrist, forearm or shoulder.

**Conclusions:**

Deep phenotyping of FTSDma reveals similarities in dystonic phenotype between instrument classes, likely related to shared technical demands, and unexpected similarities between other forms of task-specific upper extremity dystonia. A network model to explain these findings is proposed.

## Introduction

Focal task-specific dystonia of the musicians’ arm (FTSDma) is an unusual and often professionally disabling disorder of motor control. Usually beginning in the fourth decade, this painless selective impairment of the ability to play a musical instrument was first described more than a century ago by Gowers, Hammond and Duchenne [1]. FTSDma affects as many as 2% of professional musicians, including legendary musicians such as Robert Schumann, Gary Graffman, Leon Fleisher and Yehudi Menuhin [2]. Similar forms of task-specific dystonia may affect professional athletes in sports such as golf, baseball, ping pong, archery and pistol shooting [3]. These disorders (henceforth referred to as “Other Dystonia” (OD)), bear similarity to the most common form of task-specific dystonia seen by movement disorder neurologists, writer’s cramp (WC) [4].

Modern interest in FTSDma was stimulated by prominent musicians’ stories of FTSDma in the early 1980s (Gary Graffman) and later efforts to educate a broader audience about the challenges facing affected musicians (Leon Fleisher). Their efforts helped stimulate development of the field of performing arms medicine, and clinical series of patients with FTSDma in the literature soon followed. Newmann and Hochberg were the first to call attention to a relationship between the pattern of dystonia and the mechanical demands of the performer’s instrument [5] suggesting an important role for environmental influence in the development of the dystonic phenotype. Numerous studies have used neurophysiology and imaging techniques to demonstrate an important role for aberrant plasticity and impaired surround inhibition in FTSDma and WC [6,7,8]. Early imaging studies using fMRI and MEG suggested abnormalities in the somatotopic organization of the motor and sensory homunculus in FTSDma patients [9], however more recent work has called this into question [10]. Several models of FTSDma have been proposed [11,12,13], and impairments in sensorimotor processing likely play an important role.

We reviewed patients evaluated and videotaped by one neurologist in clinical practice over 25 years, to explore four questions:

- Is there a relationship between the demands of instrumental performance and the specific dystonic phenotype?
- If so, does this relationship also apply to patients with WC and OD?
- Are these findings seen in similar patients reported in the literature?
- Can deep phenotyping of FTSDma offer insights into the anatomy and biologic mechanism of these unusual disorders?

## Methods

Over 25 years, detailed records of the author’s clinical practice evaluation of 173 patients with FTSDma, 50 patients with WC, and 16 patients with OD were reviewed. 148 videos (86%) were reviewed of FTSDma, 31 (62%) of WC, and 14 (88%) of OD, for a total of 193 videos (81%), and 61 of the FTSDma patients were described in a prior paper [14]. Most patients were videotaped, always with a signed institution-approved written informed consent allowing presentation and publication of videos in scientific format. Videos were carefully analyzed at normal and ¼ speed to ensure that the initial clinical impression of dystonic phenotype was correct. Age at onset of dystonic symptoms and evaluation in clinic, instrument(s) played, and any history of peripheral trauma were querried, and a complete neurologic examination was performed. We excluded patients with a history of exposure to dopamine receptor blockers, a known family history of an identified genetic form of dystonia, or any abnormalities on neurologic examination besides dystonia. We also excluded patients with primary writing tremor and task-induced tremor linked to instrumental performance, due to the unsettled question whether this entity is a form of essential tremor, of dystonia, or a separate condition. Although not systematically assessed in every patient, the presence of mirror dystonia, sensory geste or glove effect (improvement of dystonia while wearing a disposable latex glove) was recorded. History and video documentation of spread of dystonia to other arm tasks (task-spread) or other body areas (anatomic-spread) was also noted. We defined anatomic spread as spread of dystonia outside of the affected arm to another body region.

**Figure 1:**
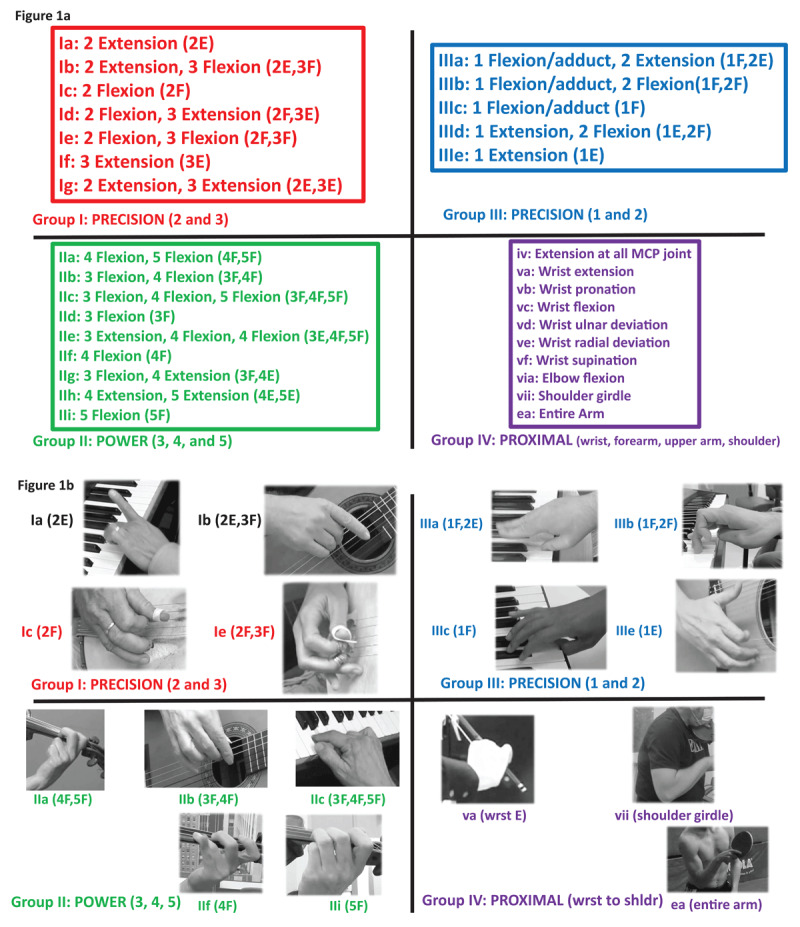
The four phenotypes of FTSDma are summarized in **Figure 1a** and **1b**. Within each group (I through IV), we assigned patterns to specific combinations of movement, using a letter (a–i), attempting to use letters earlier in the alphabet for more commonly seen phenotypes. In **Figure 1a**, group I (precision grip involving the second and/or third finger) occurs in seven phenotypes, Ia–g, involving various combinations of flexion and extension. Group II refers to the power hand, involving combinations of the third, fourth and fifth finger (nine phenotypes, IIa–i). Group III (precision grip involving the thumb and/or the second finger) occurs in five phenotypes (IIIa–e). Group IV involves various proximal parts of the arm (ten phenotypes). We subdivided the complex phenotype of proximal dystonia of Group IV into ten phenotypes (IV—iv, v(a–e), vi(a), vii and ea). Still photographs from videos of examples of representative phenotypes appear in **Figure 1b**.

After careful review, the author assigned each patient’s dystonic pattern to one of four categories (Figure 1): **group I**—involvement of the second (2) and/or third (3) finger; **group II**—involvement of the third (3), fourth (4) and/or fifth (5) fingers; **group III**—involvement of the thumb (1), second (2) and/or third (3) finger; and **group IV**—involvement of the wrist, forearm, upper arm, shoulder, or entire arm. These categories emerged as the most effective way to describe patients’ movements, as well as from the established concept of the “precision grip” vs. the “power grip”: (groups I and III involve the “precision grip” while group II involves the “power grip). These terms were coined by the English surgeon Napier in the mid-twentieth century, to refer to the hand’s capacity to grasp and manipulate objects with exceptional subtlety (precision grip), as well as to exert force on a stick or implement (power grip) [15]. Careful attention was paid to distinguish primary dystonic movements from compensatory movements. We employed examination techniques such as testing performance using multiple technical demands, observing for mirror dystonia, and applying gentle counterpressure or removable splints to specific muscle groups to help determine if removing a movement component improved or worsened performance. Complex dystonic movements were defined by their individual components (Figure 1). For each of the five families of instruments encountered (keyboard, plucked string, percussion, string and woodwind), and for WC and OD, the most common dystonic phenotypes were selected and mapped using a “heat map” approach to allow comparison between groups. We then conducted a literature search using PubMed, with key words “dystonia, music, instrument, musicians’ cramp”, netting 50 papers (references 17–66). We included all patients reported in these papers with idiopathic musicians’ dystonia, writer’s cramp or other dystonia, applying the same inclusion and exclusion criteria we used for our patient cohort (i.e. no evidence of genetic, structural or other cause). We included patients where information on instrument or task affected, hand affected, age and gender were available. Detailed descriptions of dystonia phenomenology were surprisingly sparse, and as there were no other published videos, we were unable to directly observe and analyze the movements as we did in our patient cohort.

## Results

We will first review the demographic and clinical features of our patients (173 FTSDma, 50 WC, 16 OD), and then compare the most common dystonic phenotypes of each patient group with those published in the literature.

### Demographics and Clinical Features

**Keyboard:** Demographic and clinical features of 48 keyboard instrumentalists (47 pianists and one accordion player) are summarized in **Table 1a**. 88% of patients were men, and dystonia affected the right hand in 65%, left in 25%, and bilateral in 10%. Age at onset of dystonic symptoms (by history) was 41.3 yrs, and age at evaluation by the author was 48.3 yrs. Only 15% of pianists reported a trigger (peripheral trauma or injury) directly preceding the development of dystonic symptoms. Geste maneuvers that improved dystonia and mirror dystonia were uncommon. 40% of patients experienced spread of dystonia to other tasks (usually typing) or to posture or rest. Anatomic spread of dystonia was uncommon, seen in only 10% of patients.

**Plucked strings:** Demographic and clinical features of 40 plucked string instrumentalists (32 guitar, 4 banjo, and one each of bass guitar, Koto, harp and guitar/banjo hybrid) are summarized in **Table 1b**. 87.5% of patients were men, and dystonia affected the right hand in 67.5%, left in 27.5%, and bilateral in 5%. Age at onset was 38.7 years, and age at evaluation by the author was 47.6 years. Only one patient reported a trigger (switching the size of their instrument) prior to developing dystonia. Mirror dystonia was uncommon, but a prominent and immediate improvement in dystonic movements when patients played while wearing a latex glove (the “glove effect”) [16] was seen in 10%. 35% of patients experienced spread of dystonia to other tasks (usually typing), or to posture or rest. Anatomic spread of dystonia was rare, seen in only one patient.

**Percussion:** Demographic and clinical features of 15 drummers (13 using sticks or mallets, 2 using hands) are summarized in **Table 1c**. 93% of patients were men, and dystonia affected the right hand in 40% and the left hand in 60%. Age at symptom onset was 43.8 yrs and age at evaluation by the author was 47.3 yrs. Only 20% of patients reported a trigger preceding the development of dystonia. A geste maneuver occurred in one patient, and mirror dystonia was absent. 33% of patients experienced spread of dystonia to other tasks, and anatomic spread was absent.

**Woodwind:** Demographic and clinical features of 40 woodwind instrumentalists (14 flautists, 13 clarinetists, 3 saxophonists, 3 bagpipe players, 3 woodwind doublers (those playing multiple woodwind instruments), two trumpeters, one bassoonist and one bass clarinetist) are summarized in **Table 1d** (we chose to include two trumpeters even though they are brass instrumentalists due to the similarity in their phenotype). 60% of patients were men, and dystonia affected the right hand in 42.5%, the left hand in 47.5%, and 10% bilateral. Age at symptom onset was 36.9 yrs and age at evaluation by the author was 42.8 yrs. Only 10% of patients reported a trigger preceding development of dystonia. Geste maneuvers and mirror dystonia were rare. 15% of patients experienced spread of dystonia to other tasks, and anatomic spread was rare, occurring in 10%.

**Strings:** Demographic and clinical features of 30 string instrumentalists (26 violinists, 3 violists and 1 cellist) are summarized in **Table 1e**. 50% of patients were men; 27% involved the right arm (the “bow arm”) and 73% the left arm. Age at symptom onset was 33.7 years and age at evaluation by the author was 40.8 years. 13% of patients reported a trigger preceding development of dystonia. Geste maneuvers and mirror dystonia were rare. 13% of patients experienced spread of dystonia to other tasks, and anatomic spread was not seen.

**Writer’s Cramp:** Demographic and clinical features of 50 patients with WC seen during the same period are summarized in **Table 2a**. 40% of patients were men, and 82% involved the right hand. Age at symptom onset was 37.3 years, and age at evaluation by the author was 48.5 years. Only 6% of patients reported a trigger preceding the development of dystonia. Geste maneuvers were present in 14%, and 46% had mirror dystonia when writing with the non-dominant hand. 42% of patients experienced spread of dystonia to other tasks (typing and other hand tasks), posture and rest. Only 4% of patients experienced anatomic spread of dystonia.

**Other Dystonia:** Demographic and clinical features of 16 patients with other forms of arm dystonia are summarized in **Table 2b**. 69% of patients were men, and 56% involved the right hand, 25% the left hand, and 19% bilateral. Age of symptom onset was 36.8 years, and age at evaluation by the author was 43.5 years. Only one patient reported a trigger preceding the development of dystonia. Geste maneuvers were absent, and three patients had mirror dystonia. 62.5% of patients experienced spread of dystonia to other tasks (frequently to all tasks), posture and rest, and one patient experienced anatomic spread.

**FTSDma In the Literature:** Demographic and clinical features of patients with FTSDma reported in the literature with sufficient information to allow analysis are summarized in **Table 3**. We found 23 papers reporting 382 patients with FTSDma with full descriptions of demographic and clinical features [17,18,19,20,21,22,23,24,25,26,27,28,29,30,31,32,33,34,35,36,37,38,39], but detailed descriptions of phenomenology were available in only 104 patients. An additional four papers reported only demographic information in summary form [40,41,42,43]. Of the 382 patients, 69% were male. 74% developed dystonia in the right hand, 22% in the left, and 4% bilateral. Average age of symptom onset was 32.9 years. Of the 104 patients with available descriptions of dystonic phenomenology (discussed later), 66 were pianists, 12 plucked string players, 15 percussion and 11 woodwind players (descriptions of string players were absent).

**WC in the Literature:** Demographic and clinical features of patients with WC reported in the literature included 33 patients with full demographic and phenomenologic description [44,45,46], 276 with full demographic and partial phenomenology [47,48], and 127 with clinical features without phenomenology (**Table 4a**) [49,50,51,52,53,54,55,56,57]. For a total of 426 patients in the literature, 71% were men, and average age of symptom onset was 32.3 years. Among 299 patients, 36% displayed mirror dystonia, and anatomic spread was seen in 13%. “Simple” spread of dystonia to other tasks was reported in 70% of patients, and “dystonic” spread in 30%, although these groups overlapped, and the definitions were not clearly defined.

**OD in the Literaure:** Demographic, clinical and phenomenologic features of OD were available in 17 patients reported in the literature [58,59,60,61,62,63,64,65,66], summarized in **Table 4b**. 88% were men, and the right arm was involved in 88%. Age at symptom onset was 42.4 years. Sensory tricks and mirror dystonia were uncommon, one patient experienced spread of dystonia to another task, and no patients experienced anatomic spread.

Table 5 summarizes clinical and demographic features of our patients and patients reported in the literature together.

**Table 5:** Summary demographic features (hand affected, age at onset and evaluation, gender, trigger, spread of dystonia to other tasks or anatomic regions) are compared between the five major instrument classes, writer’s cramp, and other limb dystonia. Hand affected, age of onset and gender are compared to patients with FTSDma in the literature; age of onset, gender and spread to other tasks for WC reported in the literature; and age at onset for other dystonias reported in the literature. The right hand of keyboard and plucked string players is preferentially affected; percussion and woodwind are split evenly between the hands; and string players in the left hand more often than right. Keyboard, plucked string, drum, WC and OD experienced significant spread to other hand tasks, and overall anatomic spread was rare. Male predominance was present universally, with the except of lower occurrence in our series of WC.


TASK	HAND	AGE ONSET	AGE EVALUTION	GENDER	TRIGGER	TASK SPREAD	ANATOMIC SPREAD

**Keyboard**	65% R	41.3 y	48.3 y	88% M	15%	40%	10%

	25% L						

	10% B						

**Plucked strings**	69.5% R	38.7 yr	47.6 yr	87.5% M	2.50%	35%	2.50%

	27.5% L						

	5%						

**Percussion**	40% R	43.8 yr	47.3 yr	93%M	20%	33%	0%

	60% L						

**Woodwind**	42.5% R	36.9 yr	42.8 yr	62.55% M	10%	15%	10%

	47.5% L						

	10% B						

**String**	27% R	33.7 yr	40.8 yr	50% M	13%	13%	0%

	73%L						

**Literature FTSDma**	74% R	32.9 yr		69% M			

	22% L						

	4% B						

**WC**	82% R	37.3 yr	48.5 yr	40% M	6%	42%	4$

	18% L						

**Literature WC**		32.3 yr		71% M		30%	

**OD**	56% R	36.8 yr	43.5 yr	69% M	6%	62.50%	6%

	25% L						

	19% B						

**Literature OD**	82% R	42.4 yr					

	12% L						


### Phenomenology

**Figure 2:**
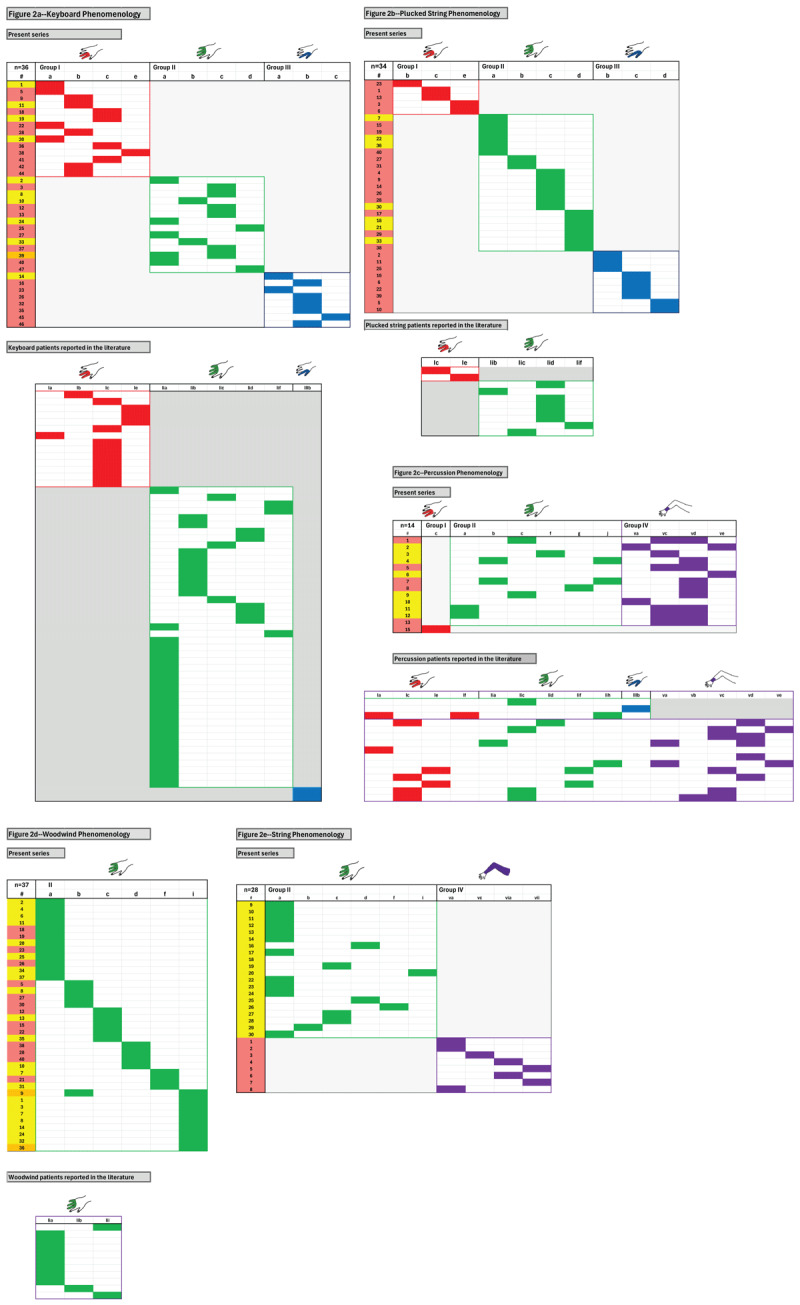
Heat maps of the most common dystonic phenotypes are presented allowing easier visual comparison between instruments and also patients reported in the literature. The group identifier appears in the top row, and individual patients are represented by a filled in rectangle in each row. Patient numbers appearing in the far-left column of each figure refer to the number assigned in the corresponding demographics table. Yellow indicates left arm involvement; salmon indicates right arm involvement; orange indicates bilateral involvement. Group I appears red, group II green, group III blue, and group IV purple. Figure 2a summarizes keyboard phenomenology, involving groups I, II and III in both the study population and the literature. Figure 2b summarizes plucked string phenomenology, with the same pattern as keyboard in Figure 1 (absent reports of group III in the literature). Figure 2c summarizes drum phenomenology, involving groups I, II and IV in our patients and the literature. Figure 2d summarizes woodwind phenomenology, involving group II. Figure 2e summarizes string phenomenology, involving group II in the left arm and group IV in the right arm in our patients (no detailed phenomenology was available in patients reported in the literature).

**Figure 3:**
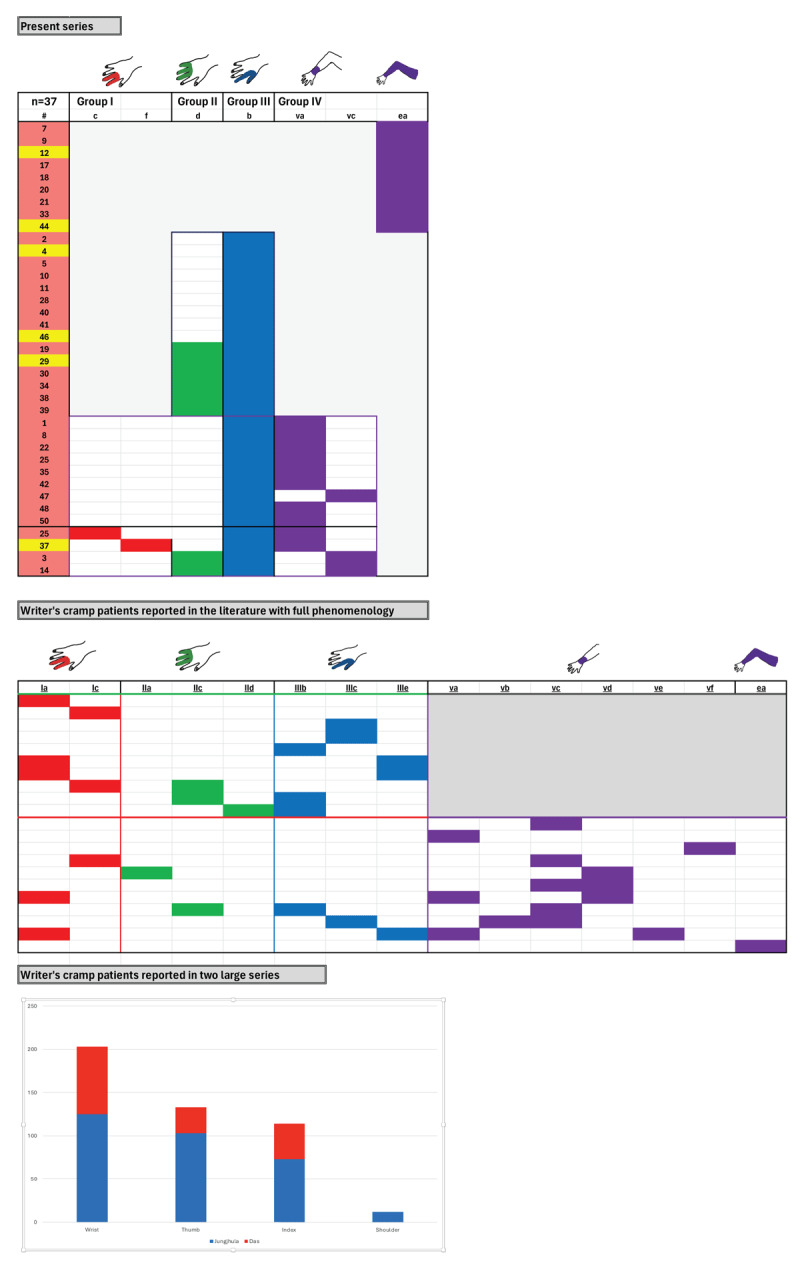
**Writer’s cramp Phenomenology.** Heat maps of the most common dystonic phenotypes in our series and in the literature are presented for patients with writer’s cramp. Within our series, patients displayed group IV (entire arm involvement), isolated group III, or combinations of group II and III, III and IV, or all four group phenotypes. In patients reported in the literature, patients presented with combinations of groups I, II and III, with and without additional the phenotype of group IV.

**Figure 4:**
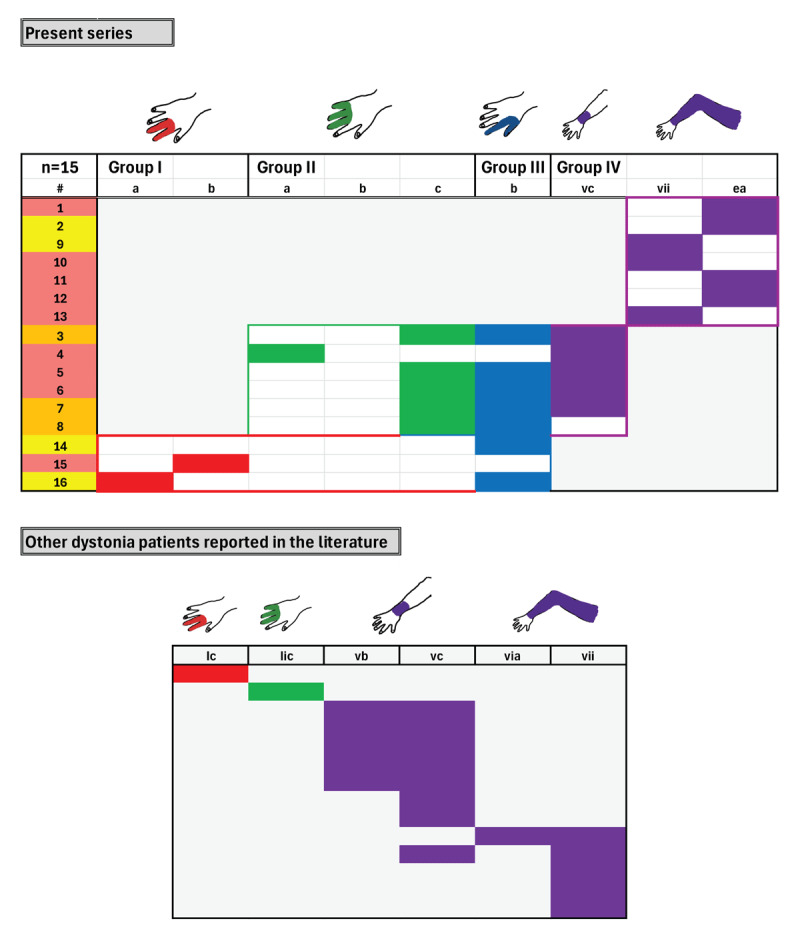
**Other dystonia Phenomenology**. Heat maps of the most common dystonic phenotypes in our series and in the literature are presented for patients with Other dystonia. Within our series, patients displayed group IV alone, group IV in combination with group III and group II, and group III in combination with group I phenotypes. Most patients presented in the literature were classified as group IV.

Figures 1a and 1b present the four major phenotypes of what we term **the dystonic repertoire** of FTSDma. **Group I** (precision grip 2, 3) involves the second and third fingers; **group II** (power grip 3, 4, 5) involves the third, fourth and fifth fingers; **group III** (precision grip 1, 2) involves the 1^st^ (thumb) and second finger; and **group IV** (proximal dystonia) involves the wrist, forearm, upper arm, shoulder girdle or entire arm. The dystonic repertoire of keyboard, plucked strings, percussion, woodwind, string, WC and OD patients seen by the author are summarized in **Tables 1a–e** and **Table 2a,b**; similar dystonic repertoire from patients reported in the literature are summarized in **Tables 3** and **4a,b**. The most common dystonic repertoire patterns for each group were tabulated, and heat maps (visually displaying the most common phenotypes) for the author’s patients in each category were compared to similar heat maps created from patients in the literature (Figures 2, 3, 4). Ten video segments (available in supplemental files) illustrate the rich phenomenology of FTSDma, WC and OD.

**Keyboard Phenomenology (Figure 2a):** Three patterns of dystonia were seen in most pianists: groups I, II and III. When the thumb, second and/or third digit were involved (groups I and III, both precision grip), the right hand was almost exclusively involved. When the power hand was involved (3, 4 and 5), dystonia was evenly split between the right and left hands. Concordance between phenotypes seen in our patients and the literature was extremely high.

**Plucked Strings Phenomenology (Figure 2b):** Three patterns of dystonia were seen in most plucked string instrumentalists: groups I, II and III. As in keyboard players, precision patterns (I and III) were limited to the right hand. Power hand (II) involvement was evenly split between right and left hand. Isolated patterns I and III were also seen in the literature.

**Percussion Phenomenology (Figure 2c):** One phenomenologic pattern was seen in most percussionists who used a stick: group II (power hand) with wrist involvement, predominantly wrist flexion (Vc) and ulnar deviation (Vd), affecting either hand. Similar patterns were seen in the literature, with the addition of the precision hand (I).

**Woodwind Phenomenology (Figure 2d):** One phenomenologic pattern was seen in most woodwind players, the power hand (II), equally split between right and left hands. A similar pattern was seen in the literature.

**String Phenomenology (Figure 2e):** Two phenomenologic patterns were seen in most string players: group II (power hand) exclusively affecting the left hand; and group IV (proximal involvement (V, Vi, VII)) exclusively involving the right arm (the “bow arm”).

**Writer’s Cramp Phenomenology (Figure 3a):** Three main patterns were seen in WC: isolated precision grip (IIIb +/– IId); precision grip plus wrist involvement (IIIb + Va or Vc); and entire arm (EA). Similar patterns were seen in patients reported in the literature.

**OD Phenomenology (Figure 4):** Aside from three patients with typing dystonia, all OD patients involved the wrist (Vc) or arm (VII, EA). A significant portion of patients with wrist flexion (Vc) also displayed flexion of all five fingers (IIc + IIIb). A similar predisposition for wrist and proximal arm was also seen in the literature.

## Discussion

We divide our discussion and analysis into three sections: Summary; Interpretation of deep phenotyping; Insights into possible mechanisms of FTSDma.

### Summary

Although this report is one of the largest and most carefully analyzed series of patients, we acknowledge several important limitations. Our patient population was subject to referral bias, likely constraining the ethnic and socioeconomic diversity of the patient population. Assessments were performed as part of routine clinical care, without the structure of a protocol that would have ensured assessment of clinical features such as mirror dystonia, geste, and glove effect in every patient. We also did not perform genetic testing to exclude known hereditary forms of dystonia; videos were taken of most but not all patients; and many patients were seen only once. While the use of a single expert examiner may be a strength, it may limit the generalizability of the methodology to a broader community of non-musician movement disorder experts. Despite these drawbacks, the similarity of clinical demographics and dystonic phenotypes of our patients to those reported in the literature suggests that our patients were likely representative.

We believe that our study offered several strengths. All patients were assessed by one examiner, trained as a professional musician and skilled in the phenomenology of dystonia. Most patients were videotaped, and each video was reviewed carefully in slow-motion to confirm the elements of the dystonic phenotype. Special attention was paid to separate primary dystonic movements from compensatory movements, a task that is often challenging. Our rationale for classifying dystonic movements into four categories employing Napier’s precision and power grip rubric may appear artificial. The decision to include the third finger in both Group 1 (precision) and Group 2 (power) may also appear artificial. However, reviewing the videos it became clear that many patients involved the third finger in precision activities, and in others, the third finger joined the fourth and fifth in the power grip. Using this rubric revealed significant patterns that mimicked clinical experience caring for these patients and correlated well with carefully phenotyped patients in the literature.

### Interpretations of Deep Phenotyping

The age of onset of dystonia onset was similar in our series and in the literature in all five instrumental groups and WC. Clinical demographics of keyboard and plucked string patients were nearly identical: an overwhelming male predominance; predominance of right-hand involvement; uncommon triggers, gestes and mirror dystonia; common spread to other tasks (particularly to typing); and uncommon spread to other body regions. We propose that the similarities in technical demands and musical requirements of these two instrument groups may have influenced a similar expression of a shared dystonic phenotype. The right hand of the piano and guitar play a primary role in setting the tempo, rhythm, harmonic and melodic structure of performance. On the piano, fingers 1, 2 and 3 have a primary influence, with 4 and 5 often playing the melodic line in the right hand, and 4 and 5 of the left articulating the baseline. In guitar, fingers 1, 2 and 3 of the right hand drive the rhythmic and melodic line. In both instruments, spread of dystonia to other tasks was common—in piano, to typing (a mechanically similar task to piano playing), and in guitar, to typing and other tasks.

Both keyboard and plucked string patients displayed common involvement of the power hand, producing patterns IIa–i. We call the over-representation of the power hand phenotype in FTSDma patients the “***power hand mismatch***”. The power hand is evolutionarily designed to stabilize the grip and exert force onto an object, rather than engaging in precision kinetics [15]. Over the last three centuries, technical demands placed on the power hand by the rapidly increasing difficulty of the musical repertoire and increasing force required to fill a 2,500-seat hall with sound may have asked the power hand (particularly the right hand of pianists and left hand of guitarists) to perform gymnastic feats for which the hand was not evolutionarily designed.

Woodwind dystonia was remarkably uniform in its phenotype, involving the power hand (group II) in combinations of adjacent fingers (4 and 5; 3 and 4), individual fingers, and all three fingers (3, 4 and 5). We note that dystonia of the power hand never skips a finger (i.e. 3 and 5 without 4). In clarinet and flute, the thumb does not press the keys, and the work of 4 and 5 is at least the equal of 2 and 3. The *power hand mismatch* may explain the predominance of this phenotype in these instruments. The technical demands of the right and left hand are similar for the clarinet, perhaps explaining why the hands are evenly affected by dystonia. In contrast, the left hand of flautists bears the sole responsibility for supporting the instrument (with the left thumb), and the left hand is also preferentially affected by dystonia. Dystonia is also seen in the left hand of string players, matching the pattern seen in woodwind players (involving the power hand in combinations of adjacent fingers (4 and 5), individual fingers, and all three fingers (3, 4 and 5)). The virtuosic violin repertoire requires the left fourth and fifth finger to bear a much greater technical burden than the second and third digits. Here, the *power hand mismatch* mimics the mechanical demands of the right hand of pianists and the left hand of plucked string players.

We note the remarkable similarity in dystonic repertoire of the right hand (“bow arm”) of string players, stick percussionists (right or left), scribing hand in WC, and affected hand in OD patients holding a club or racquet. In WC, half of the patients involved the precision hand, and the other half involved the wrist and/or the entire arm. All bow arm dystonia, stick percussion dystonia and racquet dystonia involved the wrist and/or the proximal arm. How can one explain this striking finding? We propose that holding the bow, drumstick, pen or racquet imposes a mechanical limitation in degrees of freedom of movement of the fingers and hand. This shifts the focus of motor program control to the wrist, arm and shoulder, with the hand transmitting signals from the arm rather than generating the motor phenotype. String pedagogy, drum technique, and elite tennis and golf instruction support this idea of using the proximal limb to control the stroke. We propose that these disorders represent examples of what we call “***manipulandum dystonia***”, a dystonic pattern with shared arm and hand kinetics triggered by the mechanical constraint of using the hand to hold an implement. As the predominance of the *power hand mismatch* displays the impact of instrumental performance demands in triggering dystonia, so *manipulandum dystonia* reflects the role of the arm in molding the dystonic phenotype when the hand holds a tool.

### Insights into possible mechanisms of FTSDma

Several useful models of FTSDma have been proposed [9,10,11], and space constraints prevent a detailed review and comparison in this paper. In the discussion that follows, we describe a complementary model that offers an anatomic and connectivity explanation of our description of the dystonic repertoire of FTSDma. This model also might explain the phenomena of sensory gestes, the glove effect, the long-lasting effects of botulinum toxin injections, and the potential for long-lasting remission in rare patients.

Experiments using electrical stimulation of the M1 motor cortex began more than a century ago, in higher primates (orangutans, gorillas, chimpanzees) [67], then lower primates (monkeys) [68], and eventually in man [69]. Examination of two classic papers reporting patients with intractable epilepsy or brain tumors who underwent fine mapping of the M1 motor cortex reveals unexpected similarities. Penfield’s intraoperative stimulation of the precentral gyrus (M1) in patients with intractable epilepsy of brain tumors produced activation of fingers 2–5, as well as individual finger flexion, finger extension, and activation of the thumb. A limited repertoire of grouped finger activations was also observed: 1 and 2; 2 and 3; 4 and 5; and 3, 4 and 5. Flexion movements of the fingers were more common than extension. Stimulation commonly activated the proximal arm, including wrist, elbow, and shoulder, with marked predominance of flexion over extension. While individual stimulation outputs were not reported by Penfield, the motor phenotypes triggered by direct M1 stimulation resembled the dystonic repertoire observed in our FTSD patients.

**Figure 5:**
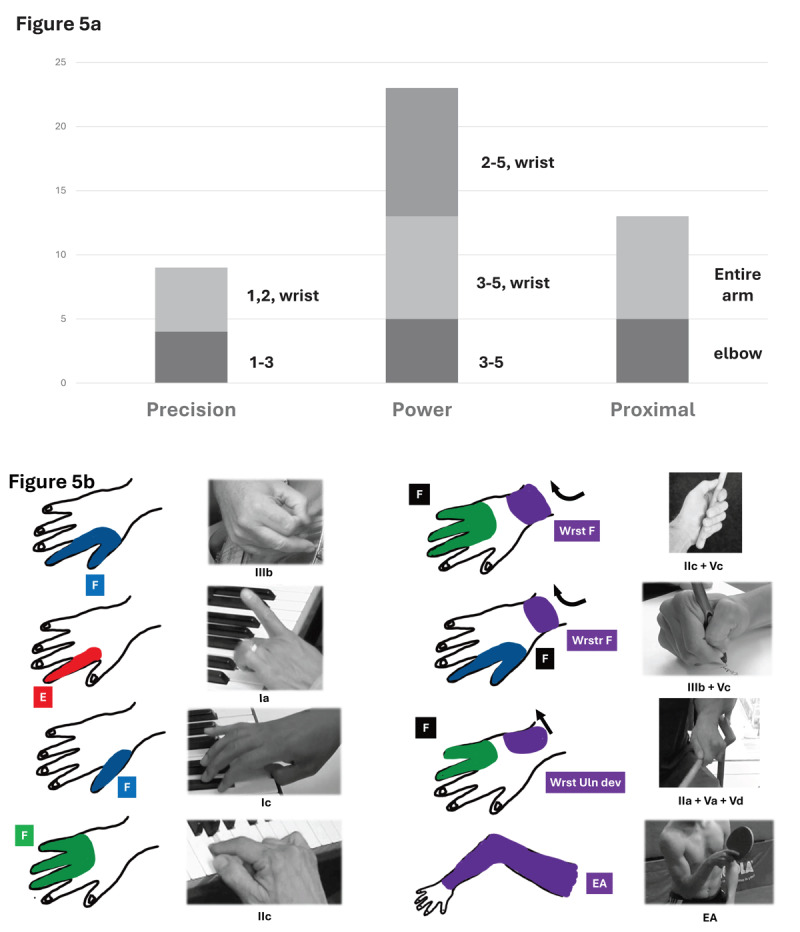
**Figure 5a** summarizes patterns of activation of the hand and arm during awake motor cortex stimulation in man [69]. Three main patterns of activation were observed: involvement of the precision hand (digits 1, 2, 3) +/– wrist; involvement of the power hand (4, 5 (+/– 3)), often with wrist involvement; and proximal arm involvement (elbow or entire arm). **Figure 5b** illustrates the striking similarity in phenotypes between Woolsey’s M1 cortical stimulation patterns (diagram of hand and arm, with precision hand in blue (flexion) or red (extension); power hand in green; and wrist to proximal arm in purple. Eight examples of patterns from the dystonic repertoire are displayed in still photos from videos, with the corresponding Woolsey picture to the left.

Even more striking, Woolsey’s detailed descriptions of fifteen patients undergoing M1 mapping prior to resection of epileptic or malignant lesions closely mimicked the dystonic repertoire we observed in FTSDma [70]. Woolsey introduced a method to summarize the results of M1 activation in lower primates [68], capturing large amounts of descriptive data in an efficient and visually compelling manner by drawing the responses during surgery. In fifteen patients undergoing mapping of the M1 cortex prior to epilepsy or tumor resection, 31 stimulations activated movements limited to the fingers, while 55 stimulations activated proximal areas of the arm (wrist, elbow, or shoulder), with or without finger activation. Three groups of stimulation patterns emerged: power grip (3, 4, 5; 3, 4, 5 + wrist; and 2–5 + wrist); precision grip (1, 2 + wrist; and 1–3); and proximal involvement (elbow and entire arm) (Figure 5a). These patterns recapitulated the dystonic repertoire observed in our patients with FTSDma, WC and OD. Further, eight patterns of activation reported by Woolsey were identical to the dystonic repertoire of FTSDma (Figure 5b).

**Figure 6:**
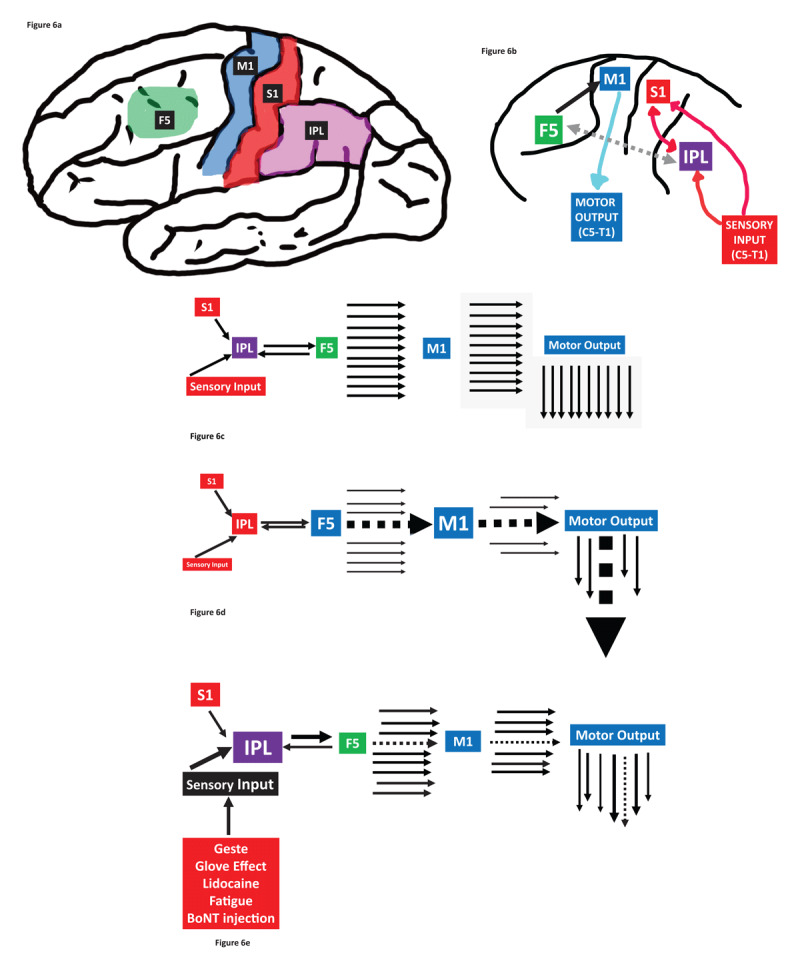
Schematics of a proposed connectivity/anatomic model of FTSDma are summarized in Figure 6. Four critical cortical areas involved in motor planning and control of the arm are outlined in **Figure 6a**: inferior parietal lobule (IPL) in light purple; M1 motor (blue) and S1 sensory (red) primary homuncular cortices; and area F5 (light green). **Figure 6b** outlines a highly simplified connectivity diagram linking these areas. Peripheral sensory input from the arm and hand (from roots C5-T1) flows to S1 and to the IPL. The IPL connects sensory input with area F5, forming a bidirectional sensory-motor planning module. Outflow from this module leaves F5 and targets the homuncular motor cortex, which sends motor control commands to motor neurons in spinal cord regions C5-T1 (after modulation by basal ganglia and cerebellar circuitry (not shown)). **Figure 6c** illustrates the normal flow of sensory-motor integration, with a well-ordered set of commands from F5 to M1, and from M1 to the anterior horn cells of C5-T1, leading to efficient and arm movement with unconstrained degrees of freedom. In a dystonic musician (**Figure 6d**), sensory input and integration of IPL with F5 is disordered. As a result, there is a loss of degrees of freedom of motor control output from F5 to M1, a predominance of one output command from M1 to the motor endplates, and a predominant dystonic phenotype that overrides the normal diversity of hand movements. **Figure 6e** illustrates the impact of treatment (e.g. geste maneuvers, glove effect, lidocaine injection, hand fatigue with exertion, or BoNT injection) on the disordered network. The effect of treatment is to partially restore the balance of sensory-motor integration, improving (to at least some extent) the degrees of freedom of commands to M1. This results in improved diversity of outputs from the M1 cortex to the anterior horn cells of C5-T1, ameliorating the monolithic dystonic phenotype and partially restoring the degrees of freedom of motor control.

We propose a novel anatomic model of FTSDma to explain the similarity in dystonic repertoire of FTSDma with Penfield and Woolsey’s results of M1 stimulation in man. The anterior inferior parietal area (AIP) and F5 supplemental motor region form a motor control system that modulates and generates commands for complex hand movements (Figures 6a,b) [71,72,73,74,75,76,77,78]. The output of this motor control network is transmitted by F5 to M1 and M1 then communicates this command through the corticospinal pathway (modulated by basal ganglia and cerebellar input) to target muscles (Figure 6c). AIP receives extensive input from primary sensory cortex (S1), Vim thalamic relay areas, as well as multi-sensory inputs.

In a normal musician, the output of F5 to M1 possesses a high degree of freedom of motor control, yielding diverse motor control commands (indicated by a low resistance setting of a filter controlling output of F5 to M1 (Figure 6c)). The “resistance filter” is proposed as a construct, a measure of the ability of F5 to send diverse command signals to M1—low resistance implies normal degrees of freedom and diversity of programming, while high resistance limits the degrees of freedom of programming commands. In a dystonic musician, the filter is aberrantly set at a high resistance level, and output from F5 activates only a small number of constrained repertoires of hand commands, resulting in a ***functional disconnection of the AIP-F5 system from M1***. The result is that the output from M1 to the effector muscles is limited to a narrow set of patterns, the dystonic phenotype, invariant and repetitive, and each attempt to use the hand to perform triggers the same limited repertoire of postures of fingers and/or proximal arm regions (Figure 6d). Application of a sensory trick such as wearing a glove [16] or long-term improvement with botulinum toxin injection resets the resistance filter, restoring the normal degree of freedom of F5’s output to M1, and therefore M1’s ability to activate a motor control output of near-infinite variability (Figure 6e).

This model proposes that FTSD is a disorder of motor programming, and that the final output mechanism (M1 to corticospinal tract outflow) remains relatively unaffected. Response to gestes and improvement with BoNT may be mediated by their effect on the AIP-F5 network’s command message to M1. Success in reprogramming the network (with either medications, toxin injections or retraining) supports the idea that FTSDma is an occupational disorder, one that is induced by arm and hand tasks that push the limits of human motor control, but with the possibility of treatment, reversal or even cure in select patients.

## Conclusions

- Deep phenotyping of FTSDma reveals that the disorder begins in the fourth decade, usually affecting the power hand in men.
- The phenotype of dystonia appears strongly influenced by the mechanical demands of specific instrumental performance, suggesting an important role for environmental (rather than endogenous) influences.
- Pathognomonic features of dystonia (mirror dystonia, sensory gestes, exquisite task-specificity, spread to other tasks, and occasionally anatomic spread) occur in musicians’ dystonia, similar to WC.
- Almost all patients with FTSDma can be classified by their dystonic phenotype into four major groups, allowing standardization of description, and aiding establishment of guidelines for treatment with BoNT injections.
- The dystonic repertoire of FTSDma resembles classic arm and hand postures generated by electrical stimulation of the primary motor cortex (M1). FTSDma may represent a dissociation or disconnection of M1 from the inferior parietal lobule (IPL) and F5 (motor control) network. This model permits an anatomic and physiologic rationale for features of FTSDma, allowing experimental study to support or refute this proposal.
- Deep phenotyping may offer a useful tool for documenting phenomenology. If this methodology can be replicated by other movement disorder clinicians, it may aid standardizing application of artificial intelligence and machine learning approaches to improve the diagnosis and treatment of FTSDma, especially in regions without ready access to expertise.

## Additional File

The additional file for this article can be found as follows:

10.5334/tohm.1044.s1 Supplemental File. Video segments 1 to 10 and Tables 1 to 4.
