# Supplementary material for: Deep Phenotyping of Musicians’ Upper Limb Dystonia
Source: Tremor Other Hyperkinet Mov (N Y). 2025 Jul 17;15:31. doi: 10.5334/tohm.1044 (PMC12273687; doi:10.5334/tohm.1044)
Supplement: Supplemental File. — Video segments 1 to 10 and Tables 1 to 4. [file tohm-15-1-1044-s1.zip › tohm-1044_frucht-s1/Table01-04-Legends.docx]

**Legend to Table 1:** Tables 1 a-e present demographic and clinical descriptions of patients with FTSDma (a: keyboard (piano and accordion); b: plucked strings (guitar, banjo, Koto, Bass guitar (bs gtr), harp); c: percussion (stick, African drum, tabla (tbl)); D: woodwind (flute, piccolo, clarinet, bass clarinet, saxophone); e: strings (violin (vln), viola (vla), cello). Presence of video documentation (video); instrument; hand affected (right, left or bilateral); age at symptom onset and evaluation (in years); gender; phenomenology pattern (presence of mirror dystonia, geste maneuvers or other notable features); trigger for dystonia; spread of dystonia to other tasks (task spread); anatomic spread of dystonia to regions outside the arm (anatomic spread); and other descriptors, were recorded for each patient. Summary totals appear in the bottom row of each table.

**Legend to Table 2:** Tables 2a and b present descriptions similar to Table 1, applied to Writer’s cramp (Table 2a) and Other dystonia (Table 2b). Presence of mirror dystonia, geste maneuvers and triggers are presented separately for Table 2a.

**Legend to Table 3:** Published reports of musicians afflicted with dystonia are summarized in Table 3. Papers with detailed phenomenological descriptions of the dystonia appear above, and papers lacking detailed description (demographic papers) follow. Limited descriptors (similar to Tables 1 and 2) appear in the columns. Full description of phenomenology was available for 104 patients, and patterns were reported using the nomenclature of this paper.

**Legend to Table 4:** Similar methodology was applied to published patients with Writer’s cramp (Table 4a) and Other dystonia (Table 4b). Published patients with Writer’s cramp were separate into those with detailed phenomenological descriptions, those with partial phenomenological report, and those with only demographic information.
