# Supplementary material for: Deep Phenotyping of Musicians’ Upper Limb Dystonia
Source: Tremor Other Hyperkinet Mov (N Y). 2025 Jul 17;15:31. doi: 10.5334/tohm.1044 (PMC12273687; doi:10.5334/tohm.1044)
Supplement: Supplemental File. — Video segments 1 to 10 and Tables 1 to 4. [file tohm-15-1-1044-s1.zip › tohm-1044_frucht-s1/Video_segments_1-10_with_legends.docx]

Due to the size of the video files, please access them from the following Google Drive when conducting your review: <https://drive.google.com/drive/folders/1Aa7QyYCrDTZyQbdI9pemTYRwFVcgQPgq?usp=drive_link>

**Video legends to segments 1-10:**

Ten video segments represent the four major phenomenology groups (segments 1-4), the rich phenomenology of FTSD (segment 5), examples of exquisite task-specificity of FTSDma (segment 6), similarity of dystonic phenotype within instrument group (segment 7), examples of the “power hand mismatch” (segment 8), “manipulandum dystonia” (segment 9), and treatment with botulinum toxin (segment 10). All patients signed institution-approved video consent allowing publication of videos in scientific format. Individual patients are referenced by instrument family (piano (Pn); plucked strings (Plc); woodwind (Wdw); strings (Str); percussion (Drm); writer’s cramp (WC); or other dystonia (OD). Patient numbers refer to tables 1 and 2. Digits are described as 1 (thumb), 2 (index), 3 (middle), 4 (ring), and 5 (pinky).

**Segment 1: Group 1--The Precision Hand—fingers 2 and 3:** A pianist (Pn30) performs a C major scale at two speeds, demonstrating isolated dystonic extension of the second digit (Ia pattern). A guitarist (Plc23) performs a G major scale using his second and third fingers, triggering dystonic extension of 2 and flexion of 3, pattern Ib. Viewing video at ¼ normal speed allows better visualization of dystonia (a strategy employed throughout these segments). A pianist (Pn41) performs an ascending scale, triggering dystonic flexion of 2 (involving the middle and distal phalanges in slow motion, pattern Ic). A pianist (Pn29) attempts an ascending C major scale, triggering dystonic flexion of 2 and extension of 3, pattern Id. Careful examination supported the assessment that extension of 3 was dystonic rather than compensatory. A banjo player (Plc3) plays a typical roll using standard picks on fingers 1-3. Although dystonic flexion of 2 and 3 appears mild (pattern Ie), the disruption in timing and rhythm of the notes is unacceptable to the performer. A traditional Irish accordion player (Pn48) demonstrates a typical tune, immediately triggering extension of 3. Finally, a guitarist (Plc20) demonstrates dystonic extension of 2 and 3 (pattern Ig), better seen in slow motion.

**Segment 2: Group II--The Power Hand—fingers 3, 4, and 5.** A violinist (Str13) displays dystonic flexion of 4 and 5 while playing a G major scale (pattern IIa). Slow-motion video demonstrates flexion of the distal and middle phalanges, and (to a lesser extent) the MCP joint. The next patient, a pianist (Pn10) experiences dystonic flexion of the left third and fourth finger (pattern IId) with preservation of normal control of the pinky. The following patient, an internationally renowned concert pianist, developed dystonia affecting fingers 3-5 after winning an international competition (Pn12). Involuntary flexion of fingers 3-5 into the palm occurs during performance of a classic Mozart sonata (pattern IIc). The following piccolo player (Wdw10) developed dystonic flexion of the left third finger at the middle phalanx (Wdw10, pattern IId). Injection of BoNT targeted at the flexor digitorum superficialis of 3 produced substantial functional improvement. The next patient, a pianist (Pn37) displays extension of 3 and flexion of 4 and 5 when playing a scale and a Chopin excerpt. The following violinist displays flexion of 4 at the MCP joint (likely lumbrical-mediated) on ascending scales, and in a passage from the Bach Chaconne. The next patient (Plc8) displays flexion of the 3^rd^ finger (likely lumbrical-mediated), pattern IIf. The following patient (Plc20) demonstrates extension of 4 and 5 while attempting to place her fingers on the fretboard. The final patient, a jazz guitarist, develops flexion of 5 (pattern IIi) immediately when he plays chords with 2, 3 and 4.

**Segment 3: Group III--The Precision Hand—fingers 1 and 2.** The first patient, Pn14, demonstrates flexion/adduction of the thumb and extension of 2 while playing (pattern IIIa). The next patient, WC40, develops flexion/adduction of the thumb and flexion of 2 while writing, producing a pincer-like posture of the thumb and index finger holding the pen (pattern IIIb). Beautiful examples of her work as a professional calligrapher prior to development of dystonia are shown. The following patient (Pn45) develops flexion of the thumb (particularly the distal phalanx) while playing (pattern IIIc). The next patient invented a hybrid banjo/guitar which he used as his main instrument for professional performance. He demonstrates extension of the thumb and flexion/adduction of 2 while playing (pattern IIId). The final patient, a banjo player (Plc3) demonstrates mild dystonic extension of the thumb while playing (pattern IIIe). Best seen at ¼ speed, the thumb over-extends while reaching for its intended target of the lowest string.

**Segment 4: Group IV—wrist, forearm, upper arm, shoulder, entire arm.** The first patient, a pianist (Pn43) develops severe isolated extension of the wrist as soon as his left hand touches the keyboard (pattern Va). The next patient (Plc34), a guitarist, developed dystonia producing pronation of the wrist, which spread to the task of writing (pattern Vb). Writing triggers pronation of the wrist as well. The following patient (OD4), a professional tennis coach, developed wrist flexion dystonia which spread to occur when he held his arms up (pattern Vc). Grasping a reflex hammer (a poor substitute for a tennis racquet) partially triggers dystonia. Dystonic flexion is better visualized at ¼ speed. The next patient, a percussionist (Drm4) develops ulnar deviation of the left wrist while playing with “soft mallets” in a fast roll (pattern Vd). Coincident extension of 5 and flexion of 3 and 4 are seen. The following patient, a violinist (Str21) demonstrates a subtle but debilitating loss of control of the speed and amplitude of the vibrato, a left arm technique critical to adding warmth and expressive nuance to the sound and musical line (VIa). Involuntary activation of the biceps produces an unwanted variance in the frequency and amplitude of her vibrato, an impairment that eventually led to professional retirement. The next patient, also a professional violinist (Str7), developed severe, devastating dystonia of the bow arm. Complex, forceful involuntary movements of the shoulder girdle and upper arm destroy his ability to control and modulate the bow’s contact with the string, the critical element of string instrumental performance (pattern VII). Although the prospect of treating such a severe dystonia appeared daunting, the patient (injected over a year by Dr. Patrick Drummond) experienced a significant improvement in control of the right arm, allowing him to reclaim an emotionally meaningful ability to make music (see video segment 10). The final patient (OD12) appears in a home video fielding ground balls at shortstop (the video was intended to showcase his skills for application to college athletic programs). Releasing the ball throwing to first base, dystonia of the throwing arm causes the ball to fly wildly off target (pattern EA, best seen in 1/10 speed slow motion).

**Segment 5: The Rich Phenomenology of FTSD.** The first patient (WC19) displays flexion and adduction of the thumb and flexion of 2 and 3 while writing with the right, dominant hand. When asked to write with the left hand, identical movements are triggered in the thumb, first and second finger (mirror dystonia). The following three patients illustrate sensory geste phenomena in FTSDma. A traditional Irish accordion player (Pn48) displays dystonic extension of 3, interrupting his ability to execute a traditional “reel” in necessary tempo and with rhythmic accuracy. Application of a plastic brace (often used by hand therapists to treat patients with rheumatoid arthritis) immediately improved dystonic extension of 3, allowing him to “bring that finger back into play”. The next patient, a blues guitarist, developed dystonic flexion of fingers 3, 4 and 5 of the right hand, preventing him from performing. He changed his playing to use a pick to pluck the strings instead of his fingers but discovered that dystonic flexion still interfered with his playing. He invented a thumb pick that allowed him to pluck the strings with the inner aspect of the thumb, a “sensory-trick-pick”. However, to be effective, the pick (worn as a ring on the thumb) needed to lightly touch the third finger. Deprived of this maneuver, dystonic flexion of 3, 4 and 5 triggers unabated. Allowing the sensory-trick-pick to touch the third finger, dystonic flexion of 3-5 is terminated. The following patient, a flautist (Wdw7) explains instrument modifications to the flute that allow her to better accommodate to dystonic flexion of 4. The next patient (WC30) demonstrates dystonic flexion and adduction of 1, and flexion of 2 and 3 while writing. Employing a sensory trick device (identical to one illustrated by Gowers in his classic chapter on writer’s cramp), dystonic movements are significantly ameliorated. The following patient, a guitarist (Plc33) eloquently explains his dystonic flexion of the left third finger. Wearing a plastic glove, dystonic flexion immediately improves, a phenomenon known as the “glove effect”. The final patient, a pianist (Pn30) demonstrates dystonic extension of the left index finger. Dystonia spread to involve typing, significantly interfering with his work. He discovered an effective sensory trick, touching the thumb to the index finger, ameliorating dystonic extension.

**Segment 6: Exquisite Task-Specificity of FTSDma.** The first patient, a prodigal classical guitarist (Plc29) achieved mastery of his instrument as a child, studying with the great guitarist Andres Segovia. He developed FTSDma of the right third finger, producing dystonic flexion triggered by playing slow ascending scales. Nevertheless, he was able to play in the traditional Flamenco style with consummate artistry without interference of dystonia. The next patient, a saxophonist (Wdw30) developed dystonic flexion of the right third and fourth fingers specifically triggered by descending scales. Ascending scales (activating extension and releaser of the fingers from the keys) was unaffected. The following three violinists demonstrate exquisite task-specificity of dystonia affecting the left hand. The left hand is responsible for changing the pitch of each note as well as the rhythm and timing, and professional violinists spend their lives training and maintaining their left hand’s ability to perform the most difficult literature of the violin repertoire flawlessly, with pure intonation and rhythm. The first of these patients (Str19), a concert violinist of renowned skill, developed dystonic flexion of the fourth and fifth fingers while playing slow scales. Paradoxically, he was able to perform four-octave scales and arpeggios (virtuosic, athletic hand performance) at high speed without impairment. The next patient developed “sticking”, persistent flexion of the third finger on the fingerboard with inability to release, specifically triggered by ascending passages in thirds (as in the opening of the Beethoven violin concerto). Dystonic over-activation of flexion of the third finger is better seen at ¼ speed. Despite this impairment, he can perform extremely difficult tasks with the left hand (Paganini 24^th^ caprice) without a problem. The final patient (Str26) can play four-octave scales without impairment. However, when demonstrating ascending scales in first position of the Bach Chaconne, dystonic proximal flexion of the fourth finger at the MCP joint interferes with performance.

**Segment 7: Similarity of Phenotypes Within Instrument:** Three flautists with strikingly similar patterns of left-hand dystonia are presented. The left hand is preferentially affected in flautists, possibly due to the increased mechanical demand of holding the instrument with the left thumb. The first patient demonstrates flexion of the middle and distal phalanges of 4 and 5 during rapid passage work. The next patient demonstrates flexion of the fourth and fifth fingers together at all three joints. The last patient demonstrates flexion of the distal fifth finger and proximal flexion of the fourth finger. The following three patients demonstrates a common dystonic pattern seen in clarinetists. The first patient demonstrates flexion at the MCP joint and extension at the middle and distal phalanges (likely lumbrical-mediated) while playing repeated patterns—this picture of “pushing the finger off the key” impairs the fine control required to ensure rapid and dexterous movements. Playing an ascending and descending E-flat major scale, the deficit may appear subtle to the examiner, but to the performer, the lack of a regular rhythmic control is unacceptable. The next patient displays a mirror image of the previous patient affecting her left hand, although milder, while playing the Mozart clarinet concerto. The final patient demonstrates the same pattern affecting the right hand, although slow-motion analysis reveals mild superimposed distal flexion of the pinky. The next three patients demonstrate a common pattern of dystonia of the wrist in drummers. The first patient develops ulnar deviation of the right hand while playing, like the pattern seen in the following patient (who also experiences “clenching” of the fourth and fifth fingers in flexion). The final patient develops a more severe pattern of wrist flexion and ulnar deviation. The final two patients (Str4 , Str6), professional violinists who played in different string quartets, developed difficulty controlling the bow arm, specifically triggered by rapid strokes in a triplet pattern. In both patients, the dystonic deficit presented as an impairment in playing the same passage, the opening of Franz Schubert’s *“Quartettsatz”*, which employs this technique. Ordinarily this rapid bowing would be handled by the wrist and fingers, however as the first patient articulates, abnormal activation and tensing of the biceps destroys his ability to control the stroke. The following patient demonstrates an identical inability to perform the same passage. Involuntary activation of her biceps is visible.

**Segment 8: The “Power Hand Mismatch”:**  Four examples of the power hand mismatch illustrate the demands placed on the fourth and fifth fingers. The first patient, Pn40, demonstrates flexion of the fourth and fifth fingers of the right hand with difficult passages. The next patient (Plc36) demonstrates severe flexion of 3, 4 and 5 when he attempts to navigate the fretboard of his guitar. The following patient (Plc2), a world-class banjo player, first demonstrates how he has re-fingered a tune, playing it with his thumb and index finger (both unaffected). Assuming the correct posture with the fifth finger planted on the face of the banjo, he engages the thumb, second and third fingers, immediately developing dystonic flexion of the third finger (and to a lesser extent, the fourth). Rhythmic accuracy and speed are significantly impaired. The last patient (Str20) displays severe dystonic flexion of 4 and 5, forcing him to struggle to extend these fingers for the next note.

**Segment 9: “Manipulandum Dystonia”:** Seven examples of manipulandum dystonia illustrate similarities in phenotype between tasks requiring the hand to grasp and control a tool (a pen, a drum stick or a violin bow). Three patients with writer’s cramp (WC35, 42 and 49) are shown. The first patient immediately develops flexion and adduction of the thumb with flexion of 2, accompanied by wrist extension. The next two patients display identical phenotypes. The third patient also triggers the phenotype when using a computer mouse. The next two patients, both percussionists (Drm6 and 15), illustrate complex dystonia associated with using a drumstick. The first patient, a Rock and Roll drummer, developed a complex, clonic dystonia affecting the pectoralis, shoulder girdle and upper and lower arm. The second patient displays tonic flexion and ulnar deviation of the right arm. The final two patients, both violinists (Str2 and 5) demonstrate bow arm dystonia. The first patient demonstrates the struggle of controlling the bow in a passage of spiccato (a technique requiring exquisite control of the right hand). The second patient demonstrates involuntary lifting of the right arm selectively on the up bow.

**Segment 10: Treatment with botulinum toxin:** Five patients treated successfully with botulinum toxin illustrate the potential for meaningful symptomatic benefit in musicians’ dystonia. The first patient (Drm15), a tabla performer, demonstrates flexion of the middle and distal phalanx of 2. Treatment with BoNT by Dr. David Simpson produced near-complete improvement of dystonia without weakness. The next three patients, treated by Dr. Patrick Drummond, experienced clinically meaningful improvement in dystonia. The first patient (Pn41), a concert pianist, developed flexion of 2 with ascending scales. Injection of flexor digitorum superficialis of 2 using high resolution ultrasound and electrical stimulation produced an excellent response, demonstrated by slow-motion video (pre-treatment on the left, post-treatment on the right). The next patient (Pn43), a pianist with severe wrist extension dystonia, is shown before and after treatment. Treatment restored his ability to effectively engage with the keyboard. The next patient Str7), a very gifted violinist, completely lost the ability to play. While initially the severity of dystonia was a cause for concern, after a year of injections; he shared a home video, illustrating the remarkable improvement in his ability to control the bow and to express emotion. The final patient (Pn17), a highly gifted pianist, developed disabling flexion of the index finger. He built a brace, which helped him to play but was only partially effective. Two weeks after injection of the flexor digitorum superficialis and profundus of 2 by Dr. David Simpson, a profound improvement in his playing was seen. He experienced a near-complete resolution of dystonia after one injection, which persisted six years later.
